# Porcine Hemagglutinating Encephalomyelitis Virus Infection *In Vivo* and *Ex Vivo*

**DOI:** 10.1128/JVI.02335-20

**Published:** 2021-05-24

**Authors:** Juan Carlos Mora-Díaz, Pablo E. Piñeyro, Rolf Rauh, William Nelson, Zianab Sankoh, Edward Gregg, José Antonio Carrillo-Ávila, Huigang Shen, Rahul K. Nelli, Jeffrey J. Zimmerman, Luis G. Giménez-Lirola

**Affiliations:** a Department of Veterinary Diagnostic and Production Animal Medicine, College of Veterinary Medicine, Iowa State University, Ames, Iowa, USA; b Tetracore, Inc., Rockville, Maryland, USA; c Andalusian Public Health System Biobank, Granada, Spain; The Peter Doherty Institute for Infection and Immunity

**Keywords:** air-liquid interface respiratory epithelial cells, betacoronavirus, CDCD (cesarean derived, colostrum deprived), coronavirus, infection, porcine hemagglutinating encephalomyelitis virus, neonatal pigs, upper respiratory tract

## Abstract

Porcine hemagglutinating encephalomyelitis virus (PHEV) is a betacoronavirus that causes vomiting and wasting disease and/or encephalomyelitis in suckling pigs. This study characterized PHEV infection, pathogenesis, and immune response in cesarean-derived, colostrum-deprived (CDCD) neonatal pigs. Infected animals developed mild respiratory, enteric, and neurological clinical signs between 2 to 13 days postoronasal inoculation (dpi). PHEV did not produce viremia, but virus shedding was detected in nasal secretions (1 to 10 dpi) and feces (2 to 7 dpi) by reverse transcriptase quantitative PCR (RT-qPCR). Viral RNA was detected in all tissues except liver, but the detection rate and RT-qPCR threshold cycle (*C_T_*) values decreased over time. The highest concentration of virus was detected in inoculated piglets necropsied at 5 dpi in turbinate and trachea, followed by tonsils, lungs, tracheobronchial lymph nodes, and stomach. The most representative microscopic lesions were gastritis lymphoplasmacytic, moderate, multifocal, with perivasculitis, and neuritis with ganglia degeneration. A moderate inflammatory response, characterized by increased levels of interferon alpha (IFN-α) in plasma (5 dpi) and infiltration of T lymphocytes and macrophages were also observed. Increased plasma levels of interleukin-8 (IL-8) were detected at 10 and 15 dpi, coinciding with the progressive resolution of the infection. Moreover, a robust antibody response was detected by 10 dpi. An *ex vivo* air-liquid CDCD-derived porcine respiratory cells culture (ALI-PRECs) system showed virus replication in ALI-PRECs and cytopathic changes and disruption of ciliated columnar epithelia, thereby confirming the tracheal epithelia as a primary site of infection for PHEV.

**IMPORTANCE** Among the ∼46 virus species in the family *Coronaviridae*, many of which are important pathogens of humans and 6 of which are commonly found in pigs, porcine hemagglutinating encephalomyelitis remains one of the least researched. The present study provided a comprehensive characterization of the PHEV infection process and immune responses using CDCD neonatal pigs. Moreover, we used an *ex vivo* ALI-PRECs system resembling the epithelial lining of the tracheobronchial region of the porcine respiratory tract to demonstrate that the upper respiratory tract is a primary site of PHEV infection. This study provides a platform for further multidisciplinary studies of coronavirus infections.

## INTRODUCTION

Porcine hemagglutinating encephalomyelitis virus (PHEV) is a large enveloped, nonsegmented, positive-sense RNA (∼30-kb) virus belonging to the family *Coronaviridae*, subfamily *Orthocoronavirinae*, genus *Betacoronavirus*, and subgenus *Embecovirus* (1; https://talk.ictvonline.org//taxonomy/p/taxonomy-history?taxnode_id=201851861). In addition to the four structural proteins common to all coronaviruses, including spike glycoprotein (S), nucleocapsid protein (N), small membrane protein (E), and transmembrane glycoprotein (M), PHEV has a layer of envelope-associated glycoproteins (hemagglutinin-esterase) that enable PHEV to hemagglutinate and hemadsorb chicken, mouse, hamster, rat, and turkey erythrocytes, a feature that differentiates PHEV from other swine coronaviruses (2–4).

PHEV infects and grows in cultures of porcine primary kidney cells (2, 5, 6), embryonic swine kidney cells (3), and adult porcine thyroid cells (7, 8), i.e., cells that are relatively easy to maintain (2, 6). In recent years, stem cell research has made significant progress toward establishing alternative *ex vivo* culture systems derived either from induced pluripotent stem cells or multipotent donor tissue stem cells (9). These systems biologically and physiologically model or mimic from which they were derived and have been proposed as alternative models for the study of infectious diseases (10).

Outbreaks of PHEV, characterized by vomiting and wasting disease (VWD) and/or encephalomyelitis in neonatal pigs (5, 11, 12), have been reported periodically since 1958 (13, 14). Although infectious for pigs of any age, PHEV produces self-limiting or subclinical infections in older pigs (15). Early studies on transmission, infectivity, and pathogenesis reproduced PHEV-associated disease under experimental conditions, including encephalomyelitis (2, 6, 16, 17) and VWD (5, 18–20) in neonatal and suckling pigs. These initial studies were performed ∼50 years ago using the scientific techniques available at the time; from the perspective of the scientific methods and technologies available at present, the PHEV infection process has not yet been fully characterized. Mouse and rat models have been commonly used to research PHEV transmission, neurotropism, and neuroinvasion (16, 21–26), but both have significant limitations given that pigs are the only species naturally susceptible to PHEV. Thus, the objective of this study was to characterize PHEV infection, pathogenesis, and immune response in cesarean-derived, colostrum-deprived (CDCD) neonatal *in vivo* (pig) and *ex vivo* (air-liquid respiratory epithelial cell culture) models.

## RESULTS

### Whole-genome sequence of PHEV 67N strain and phylogenetic analysis.

The whole-genome sequence of PHEV 67N strain was not available prior to this study. The NJ phylogenetic tree based on the complete genome sequences from the PHEV strain 67N (PHEV/67N/US/2020; GenBank accession number MW165134) assembled in this study and the 13 additional complete sequences from different PHEV isolates available in GenBank are shown in Fig. S1 in the supplemental material. The 14 sequences were grouped into two main clusters, designated PHEV-1 and PHEV-2. The PHEV/67N/US/2020 sequence was grouped into the PHEV-2 cluster, closely related to strains KY994645/CN/2008 and MF083115/CN/2014, with a 99.2% nucleotide identity with both strains, while the identity with the other 11 isolates varied from 96.0% to 98.4%.

The full-genome alignment showed that variant deletions occurred in the NS2 and the nonencoding region between the NS2 and HE genes (Fig. S3). In the corresponding position between 21515 and 22314 nucleotides (nt) for MF083115, compared to strains MF083115 and KY994645, strains KY419106, KY419107, KY419109, KY419111, and KY419113 showed a 778-nt deletion; strains KY419104, KY419105, KY419112, and KY419110 showed an 88- to 94-nt deletion; strain KY419103 showed a 13-nt deletion; and strain DQ011855 showed a 213-nt deletion. In contrast, strain 67N only showed a 2-nt deletion at position 22236 to 22237 nt compared to strain MF083115.

### Clinical signs in PHEV-infected CDCD neonatal pigs.

All pigs in the PHEV-inoculated group (*n* = 12) began exhibiting respiratory signs, including sneezing and nasal discharge on day postoronasal inoculation (dpi) 2. Fifty percent of the piglets showed transient fever (pig nos. 7, 9, 13, 14, 15, and 16), but the mean daily body temperature was not different (*P* > 0.05) between the inoculated and control groups (Fig. S2). On dpi 3, pig nos. 9, 12, 16, and 18 developed interment neurological signs, recumbency, and paddling, while pig nos. 12 and 18 showed incoordination between 8 to 13 dpi. Pig no. 8 showed inappetence on dpi 4 (Table 1). Pig nos. 8, 10, and 11 showed transient diarrhea between 4 to 8 dpi (Table 1). Pigs in the negative-control group remained clinically healthy throughout the study.

**TABLE 1:** Clinical sign detection on piglets mock or PHEV inoculated under experimental conditions

DPI^*a*^	Clinical signs in piglets (pig no.)					
	Fever^*b*^	Respiratory signs^*c*^	Neurological signs^*d*^	Inappetence	Diarrhea	Incoordination
0
1	13, 14, 15, 16
2	7, 13, 14, 16	All PHEV-inoculated pigs
3	All PHEV-inoculated pigs	9, 12, 16, 18
4	9	8	8, 10
5
6	7
7	7
8	7	11	12, 18
9	7
10	7
11
12	12
13	12
14
15

a DPI, day postinoculation.

b Fever includes piglets with body temperature higher than 40°C.

c Respiratory signs include sneezing and nasal discharge.

d Neurological signs include recumbency and paddling.

### Histologic assessment of PHEV-infected CDCD neonatal pigs.

**FIG 1:**
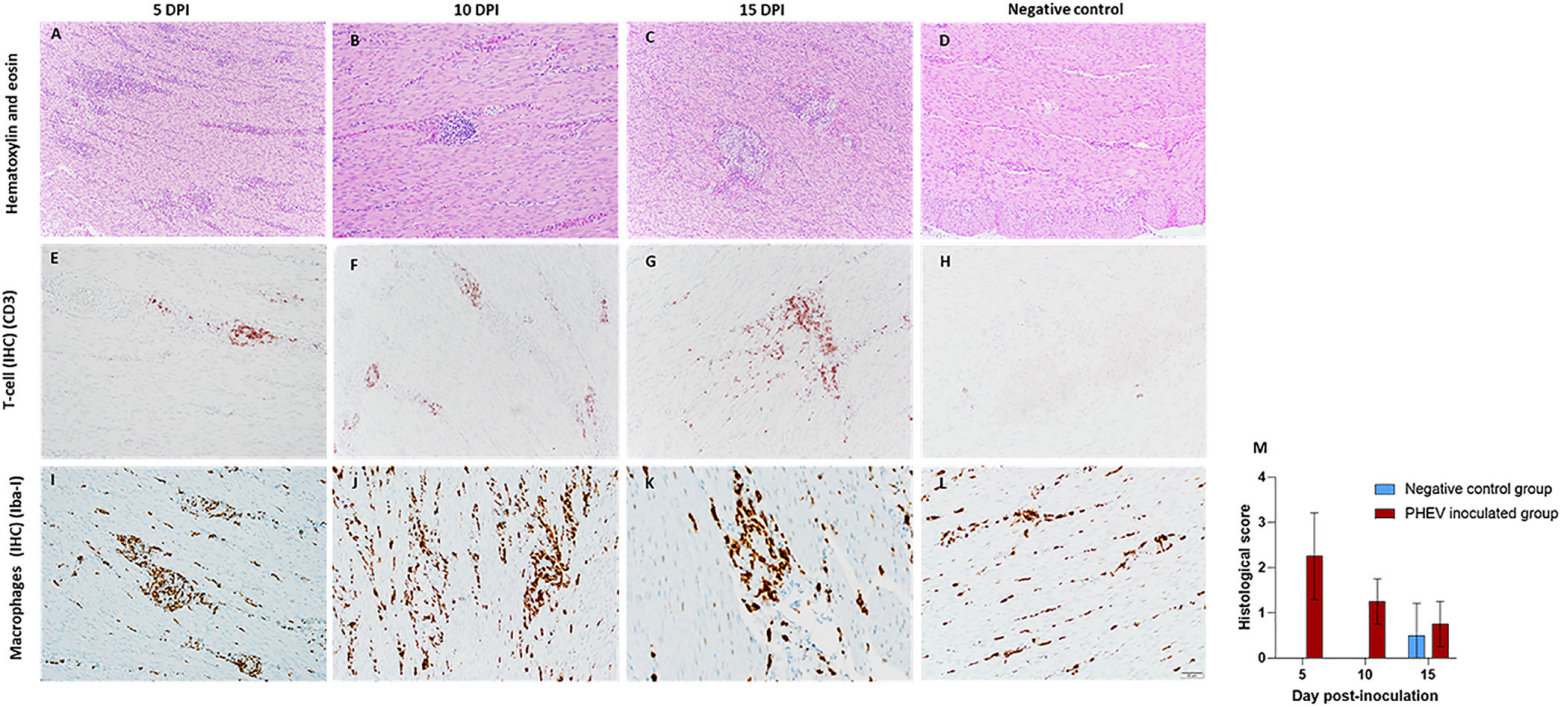
(A to D) Histological assessment of the gastric tunica muscularis from PHEV-inoculated CDCD neonatal pigs at dpi 5, 10, and 15 using hematoxylin and eosin staining. (E to L) Characterization of the inflammatory exudate using primary polyclonal antibody (pAb) antisera against CD3 diluted 1:100 (E to F) and anti-Iba-I pAb antisera at 1:500 (G to L). (D, H, L) Control tissues corresponded to specific staining. M, average score of severity of the lesions of the PHEV-inoculated group (*n* = 12) by day postinoculation. Animals with a cumulative score of 0 to 1 were considered normal, and animals with a score of ≥2 were considered positive.

Histological assessment of the full set of tissues revealed that PHEV did not induce major histopathological changes other than severe gastritis under these specific experimental conditions. Histological changes only affected the gastric tunica muscularis and were characterized by a variable degree of lymphocyte and macrophage infiltration that occasionally cuffed around small vessels and caused neuronal ganglia degeneration. On 5 dpi, 4 of 4 animals showed more severe lesions characterized by moderate, multifocal, gastritis lymphoplasmacytic, along with perivasculitis and neuritis with ganglia degeneration (tunica muscularis) (Fig. 1A). The severity of the lesions tended to resolve; hence at 10 dpi, 4 of 4 infected animals presented mild inflammatory changes (Fig. 1B), and at 15 dpi, 3 animals showed minimal inflammatory changes (Fig. 1C) that otherwise were considered within normal limits compared with control animals. The inflammatory exudate was characterized by the presence of CD3-positive T lymphocytes (Fig. 1E to G) and macrophages positive by Iba-I immunolabeling (Fig. 1I to K). However, sections were negative for CD20, a marker for B cell proliferation. The progression of the severity of the lesion is plotted in (Fig. 1M).

### PHEV RNA in nasal secretions and feces.

**FIG 2:**
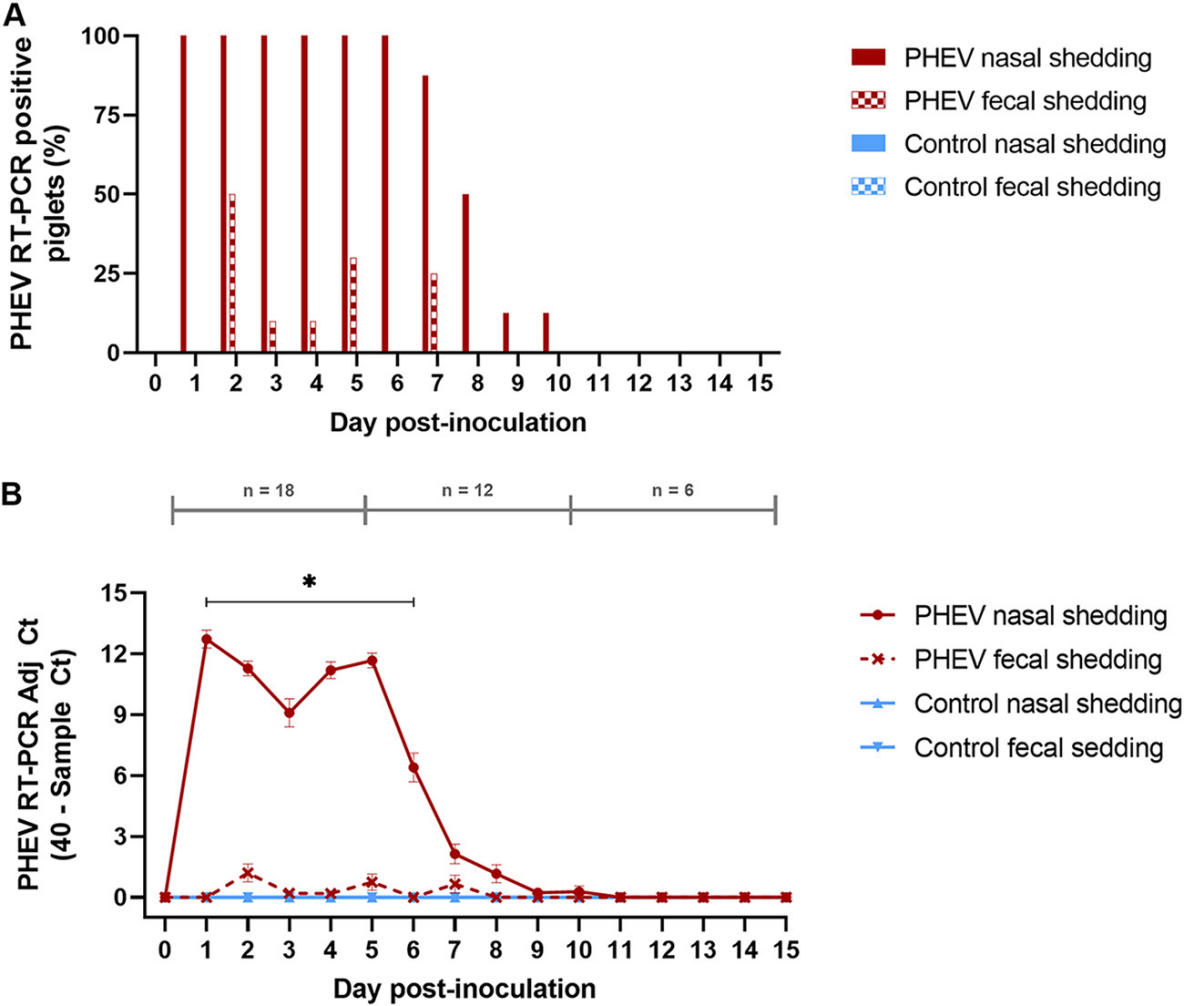
Over-time detection (15 days) of PHEV in nasal and fecal swabs by RT-qPCR, targeting the PHEV N gene, in CDCD neonatal pigs experimentally inoculated with PHEV (67N strain; *n* = 12) or mock inoculated with culture medium (control group; *n* = 6). (A) Percentage of PHEV RT-qPCR-positive piglets. (B) Mean adjusted *C_T_* (sample *C_T_*, 40) of positive samples. *, denoted statistical differences (*P* < 0.05).

Viremia was not detected in the PHEV-inoculated group at any time point during the study, but PHEV RNA was consistently detected in nasal swabs in 100% of the inoculated animals from 1 to 7 dpi (Fig. 2A) and remained detectable until dpi 10, with the highest threshold cycle (*C_T_*) values detected at dpi 1 (mean *C_T_*, 27.7; standard deviation [SD], 1.53) (Fig. 2B). Although inconsistent, PHEV RNA was also detected in rectal swabs from some inoculated piglets (pig nos. 7 to 13 and 16) between 2 and 7 dpi, with *C_T_* values peaking (mean *C_T_*, 38.80; SD, 1.56) at dpi 2 (Fig. 2A). As shown in Fig. 2B, PHEV *C_T_* values were lower in rectal swabs than nasal swabs (*P* < 0.05). No PHEV RNA was detected in samples from the negative-control group.

### PHEV RNA in tissues.

**FIG 3:**
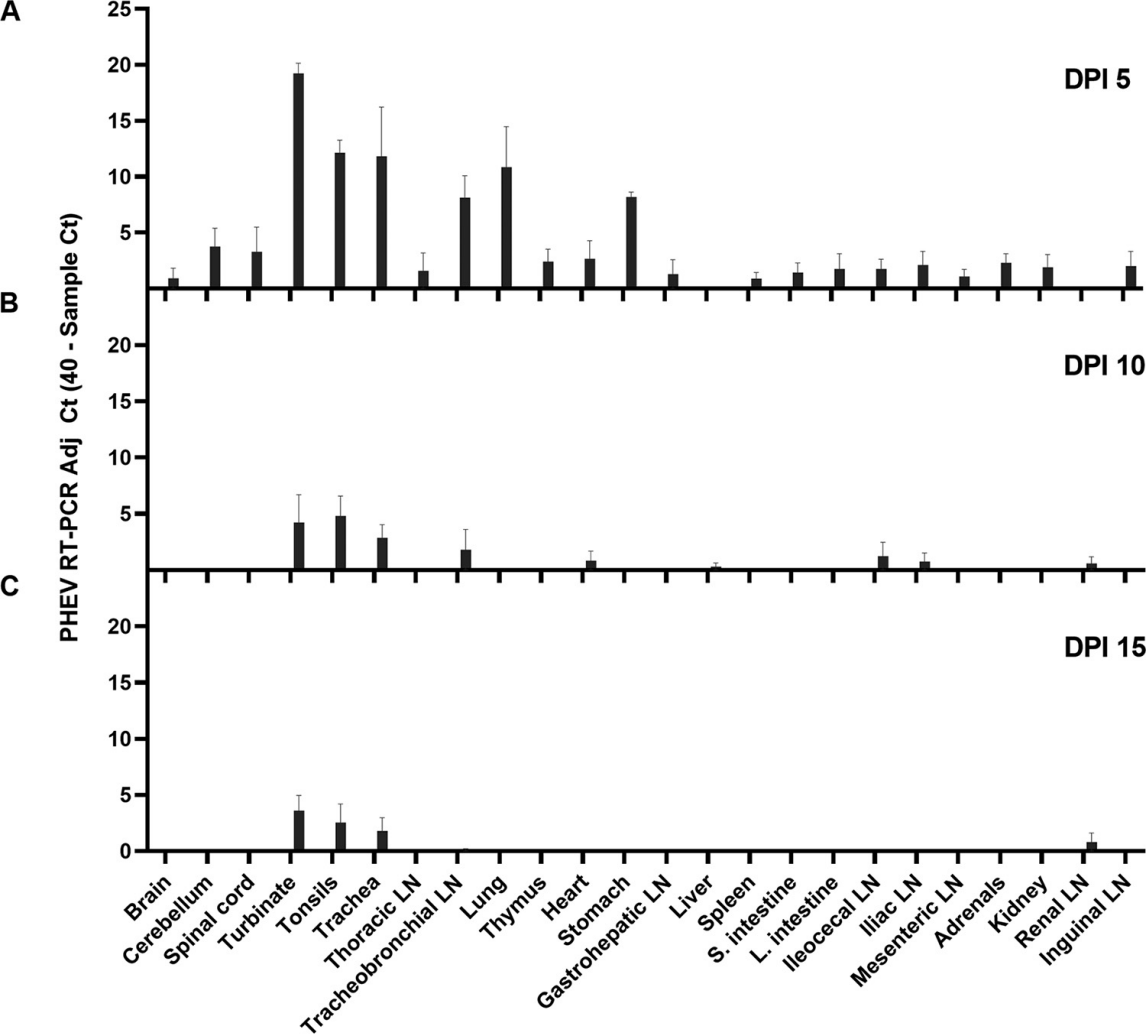
RNA detection of the virus by RT-qPCR in different tissues of CDCD piglets oronasally inoculated with PHEV (*n* = 12) after 5 (A), 10 (B), or 15 (C) days postinoculation.

The overall distribution of PHEV RNA in tissues collected at necropsy (dpi 5, 10, or 15) is given in Fig. 3 and Table 2. PHEV RNA was initially detected at various levels in all tissues of PHEV-inoculated pigs except liver, with the detection rate and RT-qPCR *C_T_* values decreasing over time. The highest concentration of virus was detected in inoculated piglets necropsied at 5 dpi in samples collected from the upper respiratory tract, i.e., turbinate (mean *C_T_*, 20.76; SD, 1.84) and trachea (mean *C_T_*, 28.18; SD, 8.82), followed by tonsils (mean *C_T_*, 27.85; SD, 2.26), lungs (mean *C_T_*, 29.14; SD, 7.25), tracheobronchial lymph nodes (mean *C_T_*, 31.86; SD, 3.92), and stomach (mean *C_T_*, 31.82; SD, 0.79). PHEV RNA was not detected in any tissues collected from piglets within the negative-control group throughout the study.

**TABLE 2:** Detection of PHEV RNA (*C*_*T*_ values) in tissues by RT-qPCR (PHEV N gene) from the PHEV-inoculated piglets

Tissue	No. positive/total no.
		Pig no. at DPI 5				Pig no. at DPI 10				Pig no. at DPI 15			
		9	10	15	16	7	11	17	18	8	12	13	14
Brain	1/12	**36.3**	ND	ND	ND	ND	ND	ND	ND	ND	ND	ND	ND
Cerebellum	3/12	**38.0**	ND	**34.3**	**32.8**	ND	ND	ND	ND	ND	ND	ND	ND
Spinal cord	3/12	ND^*b*^	**37.9**	**30.2**	**38.7**	ND	ND	ND	ND	ND	ND	ND	ND
Turbinate	9/12	**19.0**	**20.0**	**20.7**	**23.3**	**30.7**	**32.4**	ND	ND	ND	**36.6**	**33.8**	**35.1**
Tonsils	9/12	**29.3**	**26.5**	**25.4**	**30.2**	ND	**32.9**	**35.5**	**32.3**	ND	ND	**33.1**	**36.7**
Trachea	8/12	**21.9**	**21.0**	**29.8**	ND	ND	**35.7**	**34.9**	**37.9**	ND	ND	**37.6**	**35.1**
Thoracic LN^*c*^	1/12	ND	**33.6**	ND	ND	ND	ND	ND	ND	ND	ND	ND	ND
Tracheobronchial LN	6/12	**29.9**	**30.6**	**29.3**	**37.7**	ND	**32.8**	ND	ND	ND	ND	**39.6**	ND
Lung	4/12	**28.5**	**25.2**	**23.3**	**39.5**	ND	ND	ND	ND	ND	ND	ND	ND
Thymus	3/12	ND	**37.6**	**34.7**	**38.0**	ND	ND	ND	ND	ND	ND	ND	ND
Heart	1/12	ND	ND	ND	ND	ND	**36.6**	ND	ND	ND	ND	ND	ND
Stomach	3/11	**31.8**	**31.1**	-	**32.6**	ND	ND	ND	ND	ND	ND	ND	ND
Gastrohepatic LN	2/12	ND	**34.8**	ND	ND	ND	ND	ND	ND	ND	ND	ND	ND
Liver	1/12	ND	ND	ND	ND	ND	ND	ND	ND	ND	ND	ND	ND
Spleen	2/12	**38.8**	**37.6**	ND	ND	ND	ND	ND	ND	ND	ND	ND	ND
Small intestine	2/12	**36.8**	ND	ND	**37.4**	ND	ND	ND	ND	ND	ND	ND	ND
Large intestine	2/12	**38.7**	ND	**34.2**	ND	ND	ND	ND	ND	ND	ND	ND	ND
Ileocecal LN	4/12	ND	**39.0**	**35.9**	**38.0**	ND	**35.1**	ND	ND	ND	ND	ND	ND
Iliac LN	3/12	**36.1**	**35.4**	ND	ND	ND	**37.0**	ND	ND	ND	ND	ND	ND
Mesenteric LN	2/12	ND	**37.9**	**37.8**	ND	ND	ND	ND	ND	ND	ND	ND	ND
Adrenals	3/12	**37.4**	**37.0**	**36.3**	ND	ND	ND	ND	ND	ND	ND	ND	ND
Kidney	2/12	**36.2**	**36.1**	ND	ND	ND	ND	ND	ND	ND	ND	ND	ND
Renal LN	2/12	ND	ND	ND	ND	ND	**37.6**	ND	ND	ND	ND	**36.8**	ND
Inguinal LN	2/12	ND	**34.5**	ND	**37.4**	ND	ND	ND	ND	ND	ND	ND	ND

a DPI, day postinoculation.

b ND, nondetected PHEV RNA (*C*_*T*_ ≥40.0).

c LN, lymph node.

### Isotype-specific antibody response.

**FIG 4:**
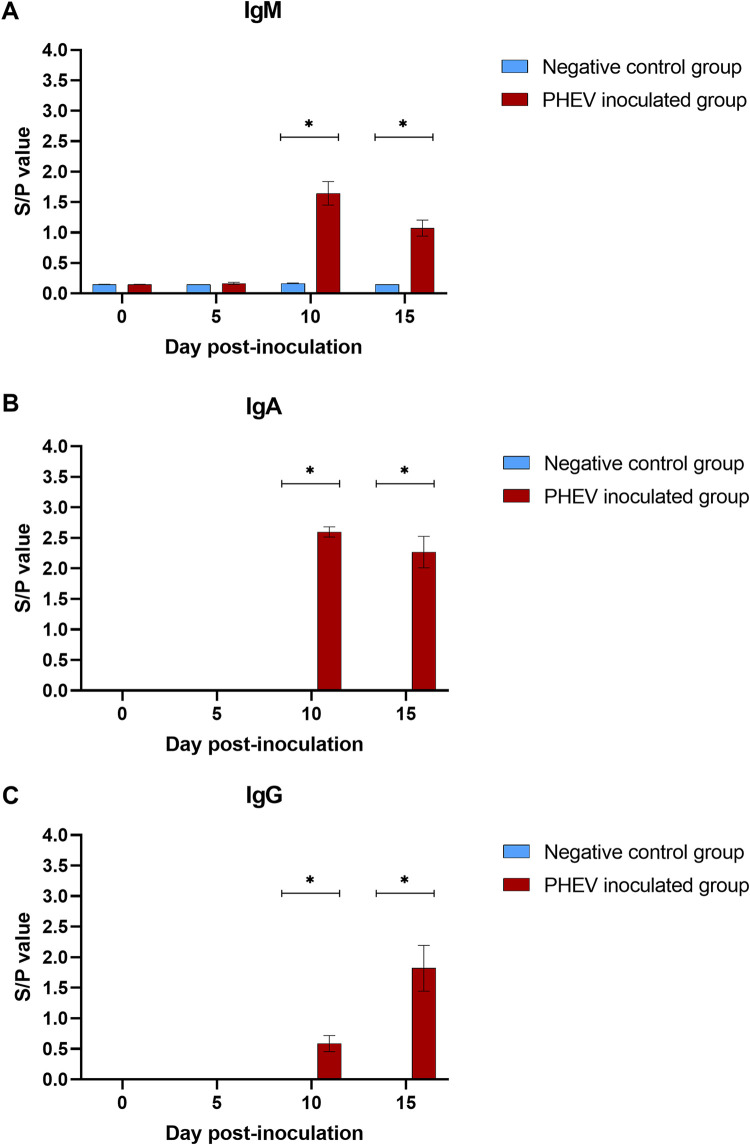
PHEV S1-based ELISA IgM (A), IgA (B), and IgG (C) responses (mean S/P values, SE) in CDCD neonatal pigs (*n* = 12) experimentally inoculated with PHEV or mock inoculated (*n* = 6) with culture medium at 0, 5, 10, and 15 days postinoculation. *, denoted statistical differences (*P* < 0.05).

The isotype-specific serum antibody response to PHEV obtained with the S1-based indirect enzyme-linked immunosorbent assay (ELISA) is presented in Fig. 4. A significant (*P* < 0.05) antibody response (IgM, IgA, and IgG) was detected in PHEV-inoculated pigs necropsied at 10 dpi (Fig. 4A to C), with declining IgM and IgA and increasing IgG levels between10 and 15 dpi (∼4-fold). Negative-control pigs remained PHEV seronegative through the study.

### Increased plasma levels of IL-8 and IFN-α in response to PHEV infection.

**FIG 5:**
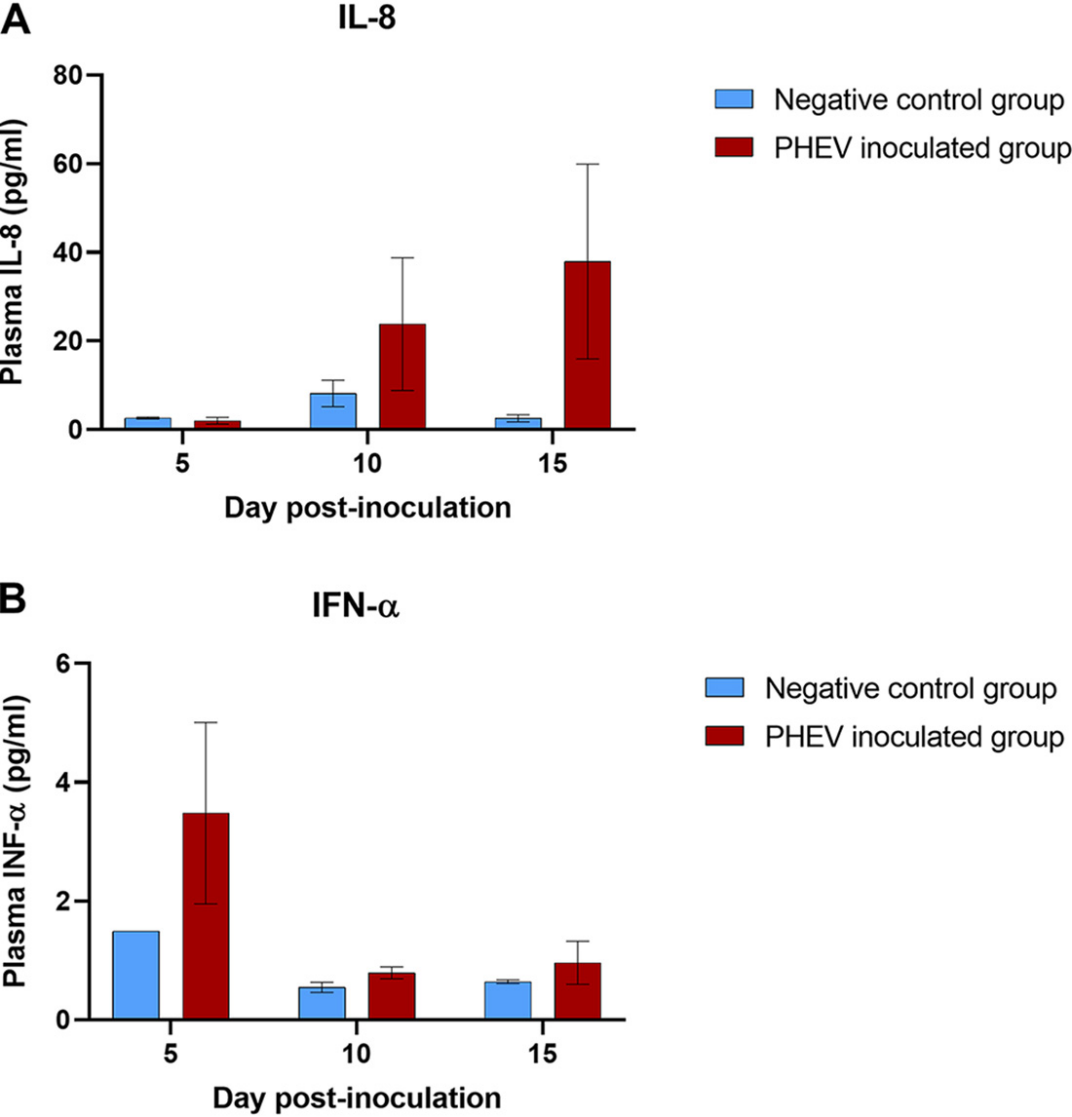
Systemic levels of IL-8 (A) and IFN-α (B) (9-plex Luminex assay; Invitrogen) in sera collected at 0, 5, 10, or 15 dpi from CDCD neonatal pigs inoculated with PHEV (*n* = 12) or mock inoculated (*n* = 6) with culture medium under experimental conditions.

Plasma samples collected on dpi 0, 5, 10, or 15 were simultaneously tested for interferon alpha (IFN-α), IFN-γ, interleukin-1 beta (IL-1β), IL-4, IL-6, IL-8, IL-10, IL-12, and tumor necrosis factor alpha (TNF-α) using a multiplex Luminex assay. Although not statistically significant, increased levels of IL-8 were detected (*P* > 0.05) in plasma from PHEV-inoculated pigs at dpi 10 and 15 compared to the negative-control group (Fig. 5A). Likewise, inoculated pigs showed increased (*P* > 0.05) plasma levels of IFN-α compared to control pigs at dpi 5 (Fig. 5B). No other remarkable changes were observed for the rest of the markers evaluated in this study.

### PHEV replication in air-liquid interface epithelial cell cultures.

**FIG 6:**
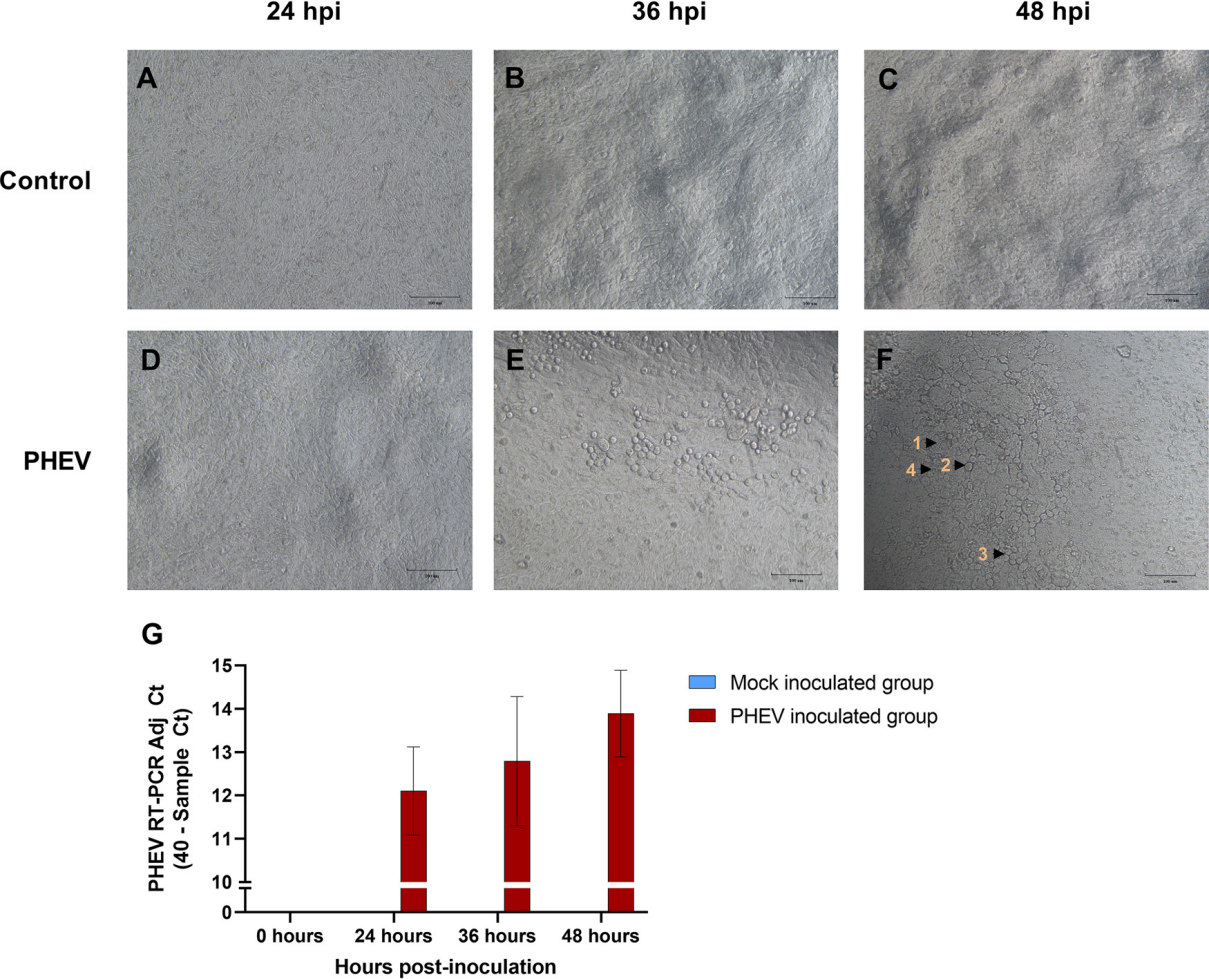
Susceptibility of air-liquid porcine respiratory epithelia culture system (ALI-PRECs) toward PHEV. ALI-PRECs (30 days old) were mock inoculated with culture medium (A, B, and C) or inoculated with PHEV (strain 67N; 128 HA titer) (D, E, and F). Cytopathic effects on PHEV-inoculated ALI-PRECs include cytoplasmic swelling and stranding (1), rounding of cells (2), cluster of rounded cells (3), and vacuolation of cells (4). (G) Detection of PHEV released into the Transwell subnatants in ALI-PRECs inoculated with PHEV using reverse transcription-quantitative PCR targeting the PHEV N gene. Representative data from two technical replicates from three biological replicates.

Differentiated air-liquid CDCD-derived porcine respiratory cells culture (ALI-PRECs) was permissive to virus entry and replication, thereby suggesting *ex vivo* that porcine respiratory epithelial cells could play an important role in PHEV infection and pathogenesis in pigs (Fig. 6). PHEV-infected cells in ALI-PRECs cultures developed marked cytopathic changes by 36 h postinfection (hpi) (Fig. 6E) that became more pronounced by 48 hpi (Fig. 6F). Cytopathic changes included cytoplasmic swelling and stranding, vacuolation, rounding of cells, clusters of rounded cells, cell shrinkage, and detachment of cells exposing the Transwell membranes. Active PHEV replication and productive infection in respiratory epithelial cells were further demonstrated by RT-qPCR; increasing levels of PHEV RNA were detected on platewell subnatants collected from infected ALI-PRECs cultures at different time points over the course of the infection (48 h) (Fig. 6G).

## DISCUSSION

The COVID-19 pandemic highlights the importance of understanding the mechanisms by which coronaviruses, even seemingly harmless species, cause disease. Although PHEV was the second coronavirus discovered (14, 27), little is known concerning viral pathogenesis and host immune response to PHEV.

A compilation of results from independent studies (1) concluded that the broad range of clinical signs (respiratory, enteric, and/or neurologic) associated with PHEV infections varied among strains isolated across countries (5, 6, 11, 28, 29) and also within the same strain (5, 19). Having said that, most of the studies conducted under experimental conditions using PHEV isolates from the United States and Japan consistently reported upper respiratory signs (17, 18, 28, 29). Similar observations were made in disease outbreak investigations (13, 29–31). In a more recent study, PHEV was linked to an influenza-like respiratory disease in show pigs in Michigan (32). In the present study, CDCD neonatal pigs oronasally inoculated with PHEV strain 67N (3, 17) exhibited mild respiratory signs and, in few cases, inappetence, transient fever, and mild diarrhea. The current scenario of increasing global spread and polymicrobial infections (e.g., coronavirus and influenza virus) complicates the recognition of pathogen-specific infections, particularly of those with no pathognomonic clinical presentation. Thus, although PHEV is currently not recognized as a respiratory pathogen, it is important to include this pathogen in the differential diagnosis of clinical outbreaks of influenza-like disease in pigs.

Although PHEV RNA was detected in all tissues collected from inoculated pigs necropsied on dpi 5, with the exception of the liver, it was detected to a greater extent in the upper respiratory tract (i.e., turbinate and trachea). Likewise, previous studies using the oronasal route of inoculation described consistent PHEV isolation from nasal mucosa and tonsils (5, 16, 19, 29). Compared to these early studies, PHEV detection across the range of tissues reported in the present study was most likely due to the use of RT-qPCR, i.e., an assay with higher analytical sensitivity than the viral isolation and/or immunofluorescence techniques used in the past.

The upper respiratory tract was earlier proposed as the natural route of infection and primary site of PHEV infection (19). Consistent with this hypothesis, an *ex vivo* ALI-PRECs model demonstrated that respiratory epithelial cells derived from the upper respiratory tract were indeed susceptible to PHEV infection and replication. Specifically, PHEV produced cytopathic changes and disruption of the ciliated columnar epithelia in the ALI-PRECs model. Similarly, Hirahara (28) observed histological changes in the mucosa of the upper respiratory tract, including degeneration of epithelial cells, loss of cilia, and slight infiltration of lymphocytes and neutrophils after 5 dpi.

As the infection progressed, significant viral load was also found in the lower respiratory tract (i.e., lungs and tracheobronchial lymph nodes) on dpi 5, which was consistent with previous studies on CDCD neonatal pigs using either oronasal (5, 16, 19, 29) or intranasal routes of inoculation (17). Mengeling et al. (3) observed pneumonia involving approximately one-third of the apical and cardiac lobes and diaphragmatic lobe, while Cutlip and Mengeling (17) observed interstitial pneumonitis of the ventral portions of the cranial lobe of the lung, lymphocytic tracheitis, and lymphocytic rhinitis. In the present study, however, no gross or histological changes were observed that suggest upper respiratory or pulmonary involvement over the course of the experiment.

Neonatal CDCD pigs shed virus in feces for ∼1 week after inoculation. A similar pattern was also observed in grower pigs under similar experimental conditions (33). However, the consistency, duration of the detection, and PHEV RNA levels were higher in nasal secretions than in feces. Appel et al. (16) first demonstrated that PHEV could be isolated from nasal secretions within the first 4 days after oronasal inoculation. Subsequent studies reported the presence of PHEV in nasal swabs from orally inoculated neonatal pigs from 1 (5) or 2 dpi (28) through 8 dpi (5, 28).

A few reports have described PHEV viremia in pigs. Andries et al. (23) reported virus isolation (4 to 6 dpi) from blood samples in 2 of 23 5-day-old pigs inoculated oronasally with PHEV (strain VW572, Belgium). Likewise, Appel et al. (16) detected viremia in 1 of 10 finisher (14-week-old) pigs inoculated oronasally with PHEV. Mora-Diaz et al. (33) detected no viremia in grower pigs inoculated under the same conditions described in this study (virus strain, dose, and inoculation route) over the course of the 42 days of experimental trial. Similarly, a previous study on bovine coronavirus, a betacoronavirus closely related to PHEV, found no viremia in naive calves naturally infected with the virus (34). Taken together, these findings suggest that viremia plays a minor role in PHEV infection and pathogenesis.

Within the body, it has been suggested that PHEV spreads from the upper respiratory and the intestinal tracts to the central nervous system (CNS) via peripheral nerves rather than the circulatory system, thereby potentially causing neurological dysfunction (19, 26). Using a combination of virus isolation and immunofluorescence antibody techniques, Andries and Pensaert determined that PHEV multiplied in the epithelium lining the respiratory tract and the tonsillar crypts, in the neuroepithelium of the nasal mucosa, and in neurons of the digestive tract to the central nervous system in 5-day-old neonatal pigs orally inoculated with PHEV (strain VW572) (23). In the present study, PHEV was detected in the spinal cord and/or cerebellum in four PHEV inoculated piglets (pig nos. 9, 10, 15, and 16) showing mild neurological signs. However, PHEV was only detected in the brain of one of these piglets (pig no. 9), an animal that also had the lowest *C_T_* values in turbinate and trachea. Interestingly, PHEV was only detected in CNS tissue at 5 dpi, coincident with the presentation of the neurological signs (i.e., paddling and incoordination) and consistent with previous reports (5, 12, 20). The lack of histological lesions poses the question of whether neurological signs were the result of virus-induced functional changes rather than inflammatory changes. To this point, Alexander et al. (13) reported that hyperesthesia was the only neurological clinical sign in piglets during field outbreaks, and the majority of the pigs that survived the first 3 to 5 days of illness recovered after 7 to 10 days. Contrarily, other studies reported encephalitis characterized by the presence of lymphocytic vascular cuffing, nodular gliosis, and neuronal degeneration in 8-day-old pigs oronasally inoculated with PHEV strain 67N (17). Likewise, only a few experimental animal studies were able to isolate virus from the brain (5, 16) and medulla (19). In the present study, PHEV was not detected in CNS tissues after 10 dpi. The time postinfection and potential mechanism of viral clearance seemed also to be an important factor for spreading and replication in the CNS and needs to be further investigated.

At this point, the neuropathogenesis of PHEV is poorly understood (35). The virus displays neurotropism and produces encephalomyelitis in mice and Wistar rats only when inoculated intracranially (21, 25, 26). A recent study based on BALB/c mice has suggested that PHEV could be used as an experimental model to investigate pathogenesis and therapies toward neurodegenerative diseases in humans (36). Our data suggested that domestic pigs (Sus scrofa domesticus) should be further investigated as a suitable animal model for PHEV-associated neurological disease because pigs are the natural host of this virus and pigs are anatomically, genetically, and physiologically more closely related to humans than other animal species (37).

In this study, CDCD neonatal pigs inoculated with PHEV 67N did not develop signs of VWD, which, based on previous reports, seemed to be strain dependent. For example, pigs experimentally infected with European strains developed VWD, but not neurologic signs (ataxia, incoordination, and/or paralysis) (5, 11) otherwise commonly associated with PHEV strains isolated in Canada (6). Mengeling and Cutlip were able to reproduce both syndromes experimentally in intranasally inoculated neonatal piglets using field isolates from naturally infected herds (20). That said, the most severe or representative microscopic lesions found in the present study were moderate, multifocal, lymphoplasmacytic gastritis with perivasculitis and neuritis with ganglia degeneration, the hallmark of VWD. However, the severity of the lesions and levels of viral RNA decreased over the 15 days of the study. Previous studies described inconsistent (19) or unsuccessful (5) PHEV isolation from the stomach of neonatal or suckling piglets. Despite evidenced neurological changes in the stomach, we did not observe clinical signs of VWD, which could be explained by the conditions under which the animals were fed compared to natural feeding, i.e., natural suction versus feeder and *ad libitum* versus time/dose controlled.

Concomitantly, a moderate inflammatory response, characterized by increased levels of IFN-α in plasma (5 dpi), and infiltration of T lymphocytes and macrophages were observed. Similar transient raising levels of IFN-α (dpi 3) were previously described in conventional grower pigs inoculated under similar experimental conditions (33). On the other hand, coinciding with the progressive resolution of the infection, increased plasma levels of IL-8 were observed at 10 and 15 dpi. IL-8 is a secretory product of stimulated macrophages and plays a fundamental role in regulating leukocyte trafficking in many infectious diseases (38). Whether or not these two events are directly related would require further investigation, even more considering the absence of inflammatory response across tissues other than the stomach.

Finally, CDCD neonatal pigs infected with PHEV developed a humoral response by 10 dpi. This response was characterized by a strong IgM, but particularly IgA antibody response, followed by a rising IgG response. A similar isotype-specific antibody response was reported in grower pigs after experimental infection with the same virus strain (7 to 14 dpi) (33). In addition, early studies reported antibody production by 6 to 7 dpi using hemagglutination inhibition (HI) assay, but its role in protective immunity remains unknown (5, 29).

This study presented a comprehensive characterization of PHEV infection *in vivo* using a refined CDCD neonatal pig model. Moreover, virus replication in the upper respiratory tract was demonstrated *ex vivo* using ALI-PRECs cultures derived from CDCD neonatal pig tracheas. ALI-PRECs systems constitute an increasingly accepted paradigm shift in disease modeling. These systems do not require live animals and are compatible with the “three Rs rules” (replacement, reduction, and refinement) of animal use (39). These models, described for different animal species (40–43), exhibit enough complexity to mimic biologically and physiologically the epithelial lining of the respiratory tract and allow the establishment of a “controlled bioassay” more representative of the whole animal than traditional culture systems. Overall, the present study provides the platform for further multidisciplinary studies toward infectious disease modeling.

## MATERIALS AND METHODS

### PHEV inoculum.

PHEV 67N, or “Mengeling strain,” originally isolated from the nasal cavity of apparently healthy swine in Iowa (1970) during a routine survey for viruses harbored in the respiratory tract, was obtained from the National Veterinary Services Laboratories (NVSL; U.S. Department of Agriculture [USDA], Ames, IA, USA) (3) was propagated in swine kidney primary (SKP) cells (NVSL) as previously described (33) and used for inoculation studies. In brief, SKP cells were maintained at 37°C with 5% CO_2_ in a 75-cm^2^ flasks (Thermo Fisher Scientific, Inc., Waltham, MA USA) with growth medium, i.e., minimum essential medium with Earle’s (EMEM) (Gibco, Thermo Fisher Scientific, Inc.) with 0.5% lactalbumin enzymatic hydrolysate (Sigma-Aldrich, St. Louis, MO, USA) supplemented with heat-inactivated 10% fetal bovine serum (FBS; ATCC, Manassas, VA, USA), 0.15% sodium bicarbonate (Sigma-Aldrich), 1% l-glutamine (Gibco, Thermo Fisher Scientific, Inc.), 1% sodium pyruvate (Gibco, Thermo Fisher Scientific), 3 μg/ml amphotericin (Gibco, Thermo Fisher Scientific, Inc.), 25 μg/ml kanamycin (Gibco, Thermo Fisher Scientific, Inc.), and 75 μg/ml gentamicin (Gibco, Thermo Fisher Scientific, Inc.). At a confluence of 80%, flasks were inoculated with 2 ml of ATV trypsin and incubated for 5 min at 37°C in 5% CO_2_; then, 5 ml of virus stock (1:128 hemagglutination assay [HA] titer) diluted 1:10 in infection medium (growth medium without FBS) was added and the flasks incubated for 4 days at 37°C in 5% CO_2_. Thereafter, the virus was harvested, titrated by hemagglutinin [HA] (33), and stored at −80°C.

### PHEV genome sequencing, assembly, and phylogenetic analysis.

The genome sequence of PHEV strain 67N was determined by next-generation sequencing. In brief, total nucleic acid was extracted from virus culture supernatant using MagMAX Pathogen RNA/DNA kit (Thermo Fisher Scientific, Inc.) as previously described (44). Double-stranded cDNA was synthesized using the Nextflex rapid RNA-Seq kit (Bioo Scientific Corp., Austin, TX USA). The sequencing library was prepared using Nextera XT DNA library preparation kit (Illumina, San Diego, CA, USA) with dual indexing. The pooled libraries were sequenced on an Illumina MiSeq platform using the 300-Cycle v2 reagent kit (Illumina) following the manufacturer’s instructions. Raw sequencing reads were preprocessed using Trimmomatic v0.36 to remove adapters and trim low-quality ends (45). Raw reads and preprocessed reads were subjected to sequencing quality analysis with FastQC (https://www.bioinformatics.babraham.ac.uk/projects/fastqc/). Cleaned reads were then fed to a comprehensive reference-assisted virus genome assembly pipeline (44, 46) with modifications. Briefly, quality-trimmed reads were mapped against the PHEV reference sequences by using BWA-MEM (47). Mapped reads were extracted by SAMtools (48) and seqtk (https://github.com/lh3/seqtk). *De novo* assembly was performed using ABySS v1.3.9 (49). The resulted contigs were manually checked and trimmed in SeqMan Pro (DNASTAR Lasergene 11 Core Suite, Madison, WI, USA).

Full-genome sequences from 13 different PHEV isolates were included in a comparative phylogenetic analysis (www.ncbi.nlm.nih.gov/nucleotide/). MEGA 7.0 was used to build the phylogenetic tree, the neighbor-joining (NJ) statistical method was used for phylogeny reconstruction, a bootstrap method with 1,000 replications was used to test phylogeny, and p-distance was selected as the substitution model.

### *In vivo* CDCD neonatal pig infection model for PHEV.

**FIG 7:**
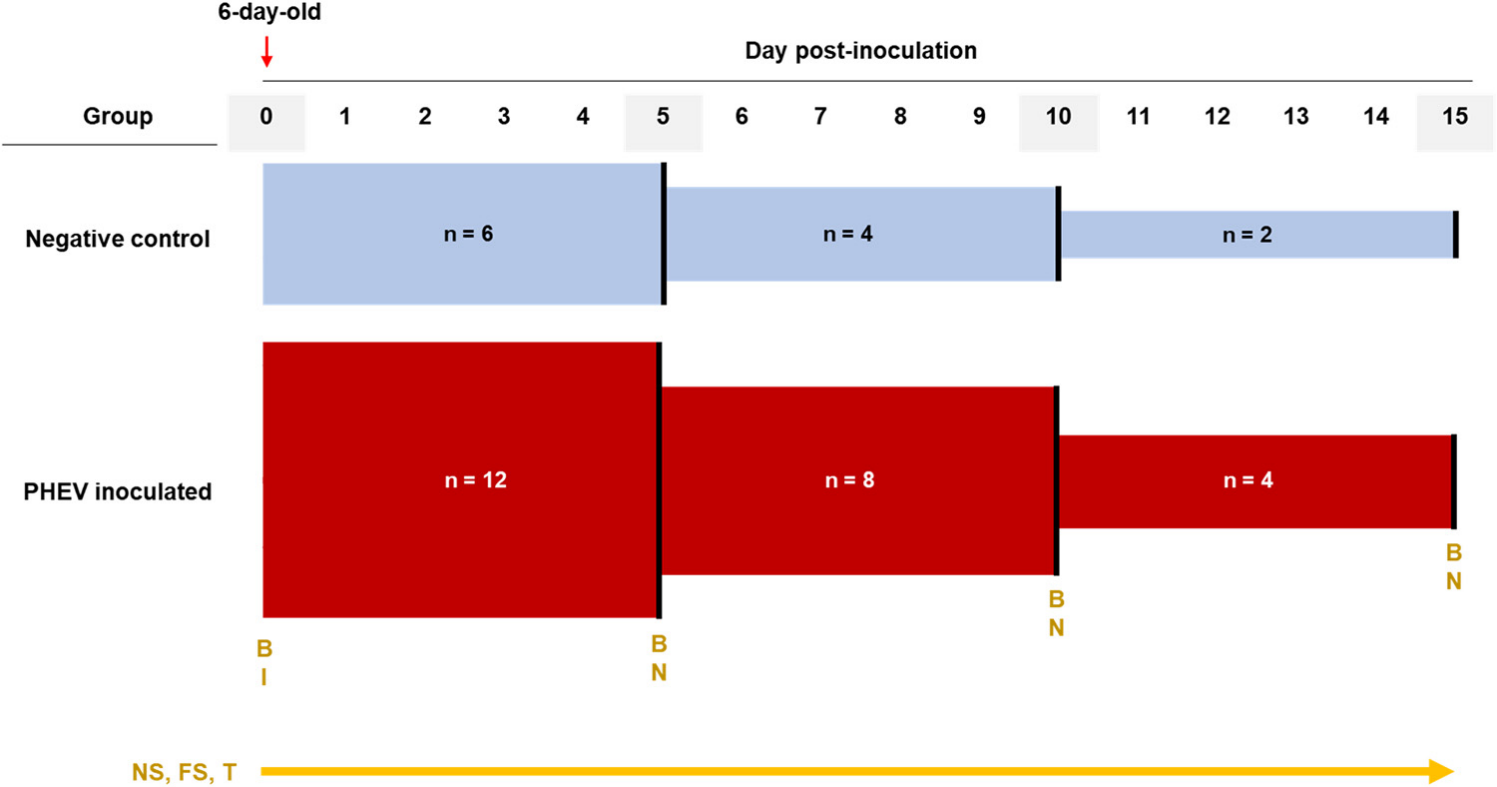
Experimental design of CDCD neonatal pigs inoculated with PHEV strain 67N (*n* = 12) or culture medium (*n* = 6). Nasal and fecal swabs were obtained daily and stored in tubes containing 1 ml of EMEM (supplemented with antibiotics). Blood samples were obtained at the beginning of the experiment and the day of the necropsy. Every 5 days, two piglets of the control group and four of the PHEV inoculated group were randomly selected for necropsy. Temperature was obtained daily. B, blood collection; I, virus inoculation; FS, fecal swabs; NS, nasal swabs; N, necropsy; T, temperature.

**(i) Animals and animal care.** The experimental protocol for the animal study was approved by the Iowa State University (ISU) Institutional Animal Care and Use Committee (IACUC; log number 12-17-8658-S; approval date, 3 January 2018). Prior to the arrival of the animals to the ISU Livestock Infectious Disease Isolation Facility (LIDIF), rooms, housing tubs, feeders, and other equipment were thoroughly cleaned, disinfected, and sanitized. Throughout the study, rooms were maintained at 34°C, and pigs were protected from direct airflow. Eighteen 6-day-old CDCD piglets (Struve Labs Inc., Manning, IA, USA) were randomly distributed into PHEV (*n* = 12) and control (*n* = 6) groups housed in two separate rooms (Fig. 7). Piglets were housed in elevated plastic tubs divided into four compartments, with one pig per compartment. Solid, clear plastic tub dividers prevented both direct contact and cross-contamination between piglets. Each compartment was equipped with a heat source, piglet nipple drinker, milk feeder, solid food feeder, rubber farrowing mat, and enrichment material (i.e., cotton rope). For the first 9 days of life, piglets were fed 150 ml of probiotic milk replacer (Struve Labs, Inc.) at 2 to 8°C three times per day in a meticulously clean feeder. When piglets were 10 days of age, milk replacer was diluted 1:1 in regular nursery purified water, and ∼237 cm^3^ (0.5 U.S. cups) of solid food (starter pellet; Heartland Co-op, Luther, IA, USA) was offered to the piglets. In addition, all piglets were intramuscularly administered 0.75 ml of vitamin E (300 IU; Vet One, Boise, ID, USA) at 10 days of age and 0.50 ml of iron (GleptoForte [gleptoferron injection], 200 mg/ml; Ceva, Lenexa, KS, USA) at 11 days of age (50–54).

**(ii) Animal inoculation and sample collection.** After 24 h of acclimation, piglets (7 days of age) in the PHEV group were inoculated with 5 ml of inoculum (0.5 ml of PHEV 67N [1:128 HA titer] in 4.5 ml of EMEM [ATCC]) oronasally (2.5 ml orally and 2.5 nasally; 1.25 ml per naris), and piglets in the control group were mock inoculated with 5 ml of EMEM (ATCC) oronasally. Piglets were clinically evaluated three times each day for general health, respiratory signs (e.g., sneezing, coughing, and fever), neurological signs (e.g., tremors, hyperesthesia, and incoordination), diarrhea, vomiting, or anorexia. Rectal temperature was measured daily.

Blood was collected from the jugular vein or cranial vena cava before inoculation and immediately prior to euthanasia (Fisherbrand, Thermo Fisher Scientific, Inc.) (Fig. 7). Blood was centrifuged (1,500 × *g* for 5 min) and serum aliquoted into cryogenic vials (Corning, Corning, NY USA) and stored at −80°C. Plasma was obtained from EDTA blood (BD, Franklin Lakes, NJ, USA) by centrifugation at 1,200 × *g* for 15 min, aliquoted into 2-ml cryogenic vials (Corning), and stored at −20°C.

Piglet nasal and rectal swabs were collected daily from day postoronasal inoculation (dpi) 0 until necropsy (Fig. 7). Immediately after collection, samples were placed in 5-ml round-bottom polystyrene tubes (Thermo Fisher Scientific, Inc.) containing 1 ml of EMEM (ATCC) supplemented with 100 IU/ml of penicillin and 100 μg/ml of streptomycin (Pen-Strep) (Gibco, Thermo Fisher Scientific, Inc.) and 0.25 μg/ml of gentamicin (Gibco, Thermo Fisher Scientific, Inc.) and then stored at −80°C.

Two piglets in the control group and four piglets in the PHEV-inoculated group were euthanized after blood collection at 5 (*n* = 6; 11 days old), 10 (*n* = 6; 16 days old), or 15 (*n* = 6; 21 days old) dpi, respectively (Fig. 7) using pentobarbital overdose and then necropsied. Fresh (stored at −80°C) and fixed (10% neutral buffered formalin) sections of the brain, cerebellum, spinal cord, turbinate, trachea, lung, stomach, small and large intestine, liver, heart, kidney, spleen, tonsils, adrenals, thymus, and lymph nodes (tracheobronchial, gastrohepatic, ileocecal, iliac, mesenteric, renal, and inguinal) were collected for viral RNA detection (PCR) and histopathological and immunohistochemical (IHC) evaluation, respectively.

### *Ex vivo* primary respiratory epithelial cell culture infection model for PHEV.

**(i) ALI-PRECs culture.** Following euthanasia, tracheal sections (below the larynx to the bronchial bifurcation) were aseptically collected from ∼10-day-old CDCD healthy control pigs and immediately placed in Dulbecco’s minimum essential/Ham’s F-12 medium with GlutaMax (DMEM/F-12) (Thermo Fisher Scientific Inc.) supplemented with Pen-Strep and 1.25 μg/ml of amphotericin B (Thermo Fisher Scientific, Inc.). Primary porcine respiratory epithelial cells (PRECs) were isolated from the tracheal mucosa as previously described (40, 41) with minor modifications. Briefly, tracheal samples were washed and incubated in phosphate-buffered saline (PBS) supplemented with Pen-Strep for 30 min at 4°C to remove blood clots. Then, samples were incubated at 4°C for 48 h in digestion medium, i.e., calcium and magnesium-free minimum essential medium (MEM; in-house), supplemented with 1.4 mg/ml pronase, 0.1 mg/ml DNase (MilliporeSigma, St. Louis, MO, USA), and 100 μg/ml Primocin (Invitrogen, San Diego, CA USA). Tissue digestion was neutralized using 10% heat-inactivated EquaFetal FBS (Atlas Biologicals, Fort Collins, CO, USA). The tissue digest containing the cells was passed through a 40-μm cell strainer, washed, pelleted, and resuspended in DMEM/F-12. The collected cells were either seeded directly using growth medium or frozen in LHC basal medium (Thermo Fisher Scientific, Inc.) containing 30% FBS and 10% dimethyl sulfoxide (DMSO) (MilliporeSigma).

Isolated PRECs were seeded at a density of ∼20,000 cells/mm^2^ on 24-well ThinCert cell culture inserts Transwell inserts (Greiner Bio-One North America Inc., Monroe, NC, USA) previously coated with collagen from human placenta (Bornstein and Traub type IV; MilliporeSigma). For the first 24 h, cells were grown at 37°C and 5% CO_2_ in growth medium (GM1) containing DMEM/F-12 supplemented with 10% FBS, 1× MEM nonessential amino acids, Pen-Strep, and 1.25 μg/ml amphotericin B. After 24 h, GM1 was removed from platewell and Transwell inserts and replaced with growth medium (GM2) consisting of DMEM/F-12 supplemented with 1,400 nM hydrocortisone, 2,700 nM epinephrine, 100 nM retinoic acid, 9.7 nM T3 (Cayman Chemicals, Ann Arbor, MI, USA), 0.5 ng/ml murine epidermal growth factor (EGF) (PeproTech, Rocky Hill, NJ, USA), 1× insulin-selenium-transferrin (Thermo Fisher Scientific, Inc.), 1× HEPES (Thermo Fisher Scientific, Inc.), 2% Ultroser-G (Pall France, Cergy, France), Pen-Strep, and 1.25 μg/ml amphotericin. GM2 medium was replaced every 2 to 3 days until PRECs were completely differentiated into air-liquid porcine respiratory epithelial cells (ALI-PRECs). On day 18 postseeding, PRECs on Transwells were completely confluent with no visible medium seepage, and a shiny glaze that resembled mucus was observable on the surface of all cultures under the microscope (Olympus CKX4; Olympus Corp., Center Valley, PA, USA). Cilia cell development and differentiation continued through days 27 to 30 (Movie S1 in the supplemental material). Upon complete differentiation, ALI-PRECs were then used for PHEV infection studies or further characterization.

**(ii) PHEV infection in ALI-PRECs.** Completely differentiated ALI-PREC cultures were inoculated with 250 μl of PHEV 67N (1:128 HA titer) diluted in infection medium DMEM/F-12 supplemented with 2% Ultroser G, 1× MEM nonessential amino acids, 1× HEPES, Pen-Strep, and 2 μg/ml *N-p*-tosyl-L-phenylalanine chloromethyl ketone (TPCK) trypsin or mock inoculated with infection medium, and incubated for 6 h at 37°C and 5% CO_2_. Thereafter, the inoculum was removed, the cultures were washed once with DMEM/F-12, fresh infection medium was added to the plate wells, and the plates were incubated 24 to 48 h at 37°C and 5% CO_2_. ALI-PREC cultures were monitored daily under the microscope (Olympus CKX4) for the presence of cytopathic changes, and subnatant samples were collected periodically to monitor virus replication.

### Pathological evaluations.

The presence of gross lesions was assessed by a veterinary pathologist at necropsy and tissue samples collected, fixed in 10% buffered formalin, and processed for routine histopathologic examination. Briefly, tissues were dehydrated, impregnated, and embedded in paraffin, sectioned at 5 μm, mounted on glass slides, and stained using hematoxylin and eosin. In addition, characterization of the inflammatory exudate was performed by immunostaining using primary polyclonal antibody (pAb) antisera against cluster of differentiation (CD) 3 diluted at 1:100 (Dako/Agilent, Santa Clara, CA, USA), rabbit anti-CD20 pAb antisera at 1:100 (Thermo Fisher Scientific, Inc.), and anti-Iba-I (macrophages/microglia) pAb antisera at 1:500 (Abcam, Cambridge, MA, USA). Using a blinded process, each tissue was evaluated for inflammatory, degenerative, or necrotic changes. Tissues with inflammatory changes were semiquantitatively scored based on the type of inflammatory process, severity, distribution, and vascular changes. The severity of lesions was scored as 0, no pathological changes; 1, tissues/inflammation lymphoplasmacytic, minimal, multifocal; 2, tissues/inflammation lymphoplasmacytic, mild, multifocal, with perivasculitis; and 3, tissues/inflammation, moderate, multifocal, with perivasculitis and neuritis with ganglia degeneration. Animals with a cumulative score of 0 to 1 were considered normal, and animals with scores ≥2 were considered positive. The average lesion score was calculated by each dpi group.

### PHEV RNA extraction.

Fecal and nasal swabs were processed by compressing the swab against the walls of the tube to elute the sample followed by centrifugation (2,500 × *g*, 5 min) to remove debris. The supernatant was then aliquoted into 2-ml cryogenic tubes (BD Falcon, Franklin Lakes, NJ, USA) for extraction or stored at −80°C. Tissues (∼1 g) were manually pureed in a homogenizer blender filter bag (Whirl-Pak; Nasco, Madison, WI, USA) containing 1 ml of UltraPure distilled water (Invitrogen). The liquid homogenate was transferred to 5-ml round-bottom polystyrene tubes (Thermo Fisher Scientific, Inc.), aliquoted (250 μl) into 2-ml cryogenic tubes (BD Falcon) for extraction, or stored at −80°C. Likewise, PHEV-infected and noninfected ALI-PRECs were collected in TRIzol reagent (Thermo Fisher Scientific, Inc.) for viral RNA isolation. Viral RNA extractions were performed using the MagMAX-96 Pathogen RNA/DNA kit (Applied Biosystems, Waltham, MA, USA) with KingFisher Flex 96 magnetic processor (Thermo Fisher Scientific, Inc.) following the manufacturer’s instructions.

### PHEV RT-qPCR.

A quantitative PHEV RT-PCR developed jointly by Tetracore, Inc. (Rockville, MD, USA) and the ISU Veterinary Diagnostic Laboratory (VDL) (33) was used to test for PHEV RNA in serum, tissues, nasal swabs, and rectal swabs. The assay targeted the conserved regions of the nucleocapsid (N) gene using a cocktail of primers and probes. In brief, each 25-μl RT-qPCR was set up by combining 19 μl of PHEV RT-qPCR master mix and 1 μl of the enzyme blend (reverse transcriptase and RNase inhibitor). An internal control (IC) was used as an extraction control, with 6 μl of the IC added to the lysis buffer. Then, 5 μl of the extracted sample RNA with IC was added to the master mix. All RT-qPCRs were performed in duplicate, and a negative extraction control (NEC), positive extraction control (PEC), and no-template control (NTC) were included in each run. All PCRs were run on a Rotor-Gene Q (Qiagen, Germantown, MD, USA) with cycling conditions 48°C for 15 min and 95°C for 2 min holding; 45 cycles, 95°C for 10 s denaturation and 60°C for 40 s amplification; and data collection. The PCR results were analyzed using Rotor-Gene Q Pure Detection software (v2.3.1), and samples with threshold cycle (*C_T_*) values >40 were considered negative.

### PHEV IgG, IgA, and IgM ELISAs.

Isotype-specific (IgG, IgA, or IgM), PHEV recombinant, S1 protein-based indirect ELISAs were used to evaluate the antibody response to PHEV infection (15). In brief, the coding region of the PHEV S1 protein was expressed in frame with the Fc portion of human IgG1 in a mammalian expression system (pNPM5 expression vector and HEK293 cells), and the soluble Fc-S1 fused protein was purified by protein A affinity chromatography (GE Healthcare, Pittsburgh, PA, USA) followed by Fc tag cleavage and further purification of PHEV S1 protein by nickel-chelating Sepharose Fast Flow affinity chromatography (GE Healthcare). PHEV S1 protein (0.47 mg/ml stock) was then coated (0.94 μg/ml in PBS, pH 7.4) onto 96-well plates (Thermo Scientific Immuno Breakables Modules; Thermo Fisher Scientific, Inc.) and incubated at 4°C for 16 h. Plates were then washed 5 times (350 μl/well) with PBST (PBS, pH 7.4, and 0.1% Tween 20), blocked with a 1% (wt/vol) bovine serum albumin solution (Jackson ImmunoResearch, West Grove, PA, USA), incubated at 25°C for 2 h, dried at 37°C for 3 h, and stored at 4°C until testing.

For testing, samples and controls (1:100; 100 μl/well) were incubated at 37°C for 1 h, plates were washed 5 times with PBST, and then 100 μl of peroxidase-conjugated goat anti-pig IgG (Fc) (1:30,000), IgA (1:2,000) or IgM (1:3,000) antibody (Bethyl Laboratories, Inc., Montgomery, TX, USA) was added. Plates were incubated at 37°C for 1 h, washed, and then 100 μl of tetramethylbenzidine-hydrogen peroxide (TMB) substrate solution was added to each well (Surmodics IVD, Inc., Eden Prairie, MN, USA). After a 5-min incubation at room temperature in the dark, the reaction was stopped by adding 100 μl of stop solution per well (Surmodics). Optical density was measured at 450 nm using an ELISA plate reader (BioTek Instruments, Inc., Winooski, VT, USA) operated with commercial software (SoftMax Pro 7; Molecular Devices, San Jose, CA USA). Antibody responses were reported as sample-to-positive (S/P) ratios.

$$\text{S/P}\;\text{ratio}=\;\frac{\text{sample OD – negative control mean OD}}{\text{positive control mean OD – negative control mean OD}}$$

where OD is optical density.

### Multiplex porcine cytokine and chemokine immunoassay.

A porcine cytokine and chemokine 9-plex Luminex assay, including IFN-α, IFN-γ, IL-1β, IL-4, IL-6, IL-8, IL-10, IL-12, and TNF-α (ProcartaPlex panel; Invitrogen, Thermo Scientific, Inc.), was performed as directed by the manufacturer on plasma samples collected from PHEV-inoculated and negative-control groups. Testing was performed using a Bio-Plex 200 system operated by the Bio-Plex Manager software (Bio-Rad, Hercules, CA, USA). The fluorescence intensity of each sample was subtracted from the blank wells, and the concentration of each cytokine was calculated from the standard curve generated from the kits’ internal standards and analyzed using GraphPad Prism 8 (GraphPad Software Inc., La Jolla, CA, USA).

### Data analysis.

Statistical analyses of the difference in detection of PHEV RNA in nasal and fecal swabs from the virus-inoculated group were performed using multiple *t* tests. The statistical significance was determined using the Holm-Sidak method, with alpha = 0.05. The evaluation of serum antibody (IgA, IgM, and IgG) levels between the PHEV- and mock-inoculated groups by dpi (5, 10, and 15) was analyzed using unpaired *t* tests. Differences in plasma cytokine levels between groups (PHEV- and mock-inoculated groups) were assessed using unpaired *t* tests. For all analyses, a *P* value of <0.05 was considered statistically significant. Statistical analyses and plots were performed using GraphPad Prism 8.

### Data availability.

A new sequence was deposited in GenBank under accession number MW165134 (hemagglutinating encephalomyelitis virus [PHEV], strain 67N, complete CDs).
