# Supplementary material for: Porcine Hemagglutinating Encephalomyelitis Virus Infection In Vivo and Ex Vivo
Source: J Virol. 2021 May 24;95(12):e02335-20. doi: 10.1128/JVI.02335-20 (PMC8316118; doi:10.1128/JVI.02335-20)
Supplement: Supplementary file 2 [file jvi.02335-20-s0002.pdf]

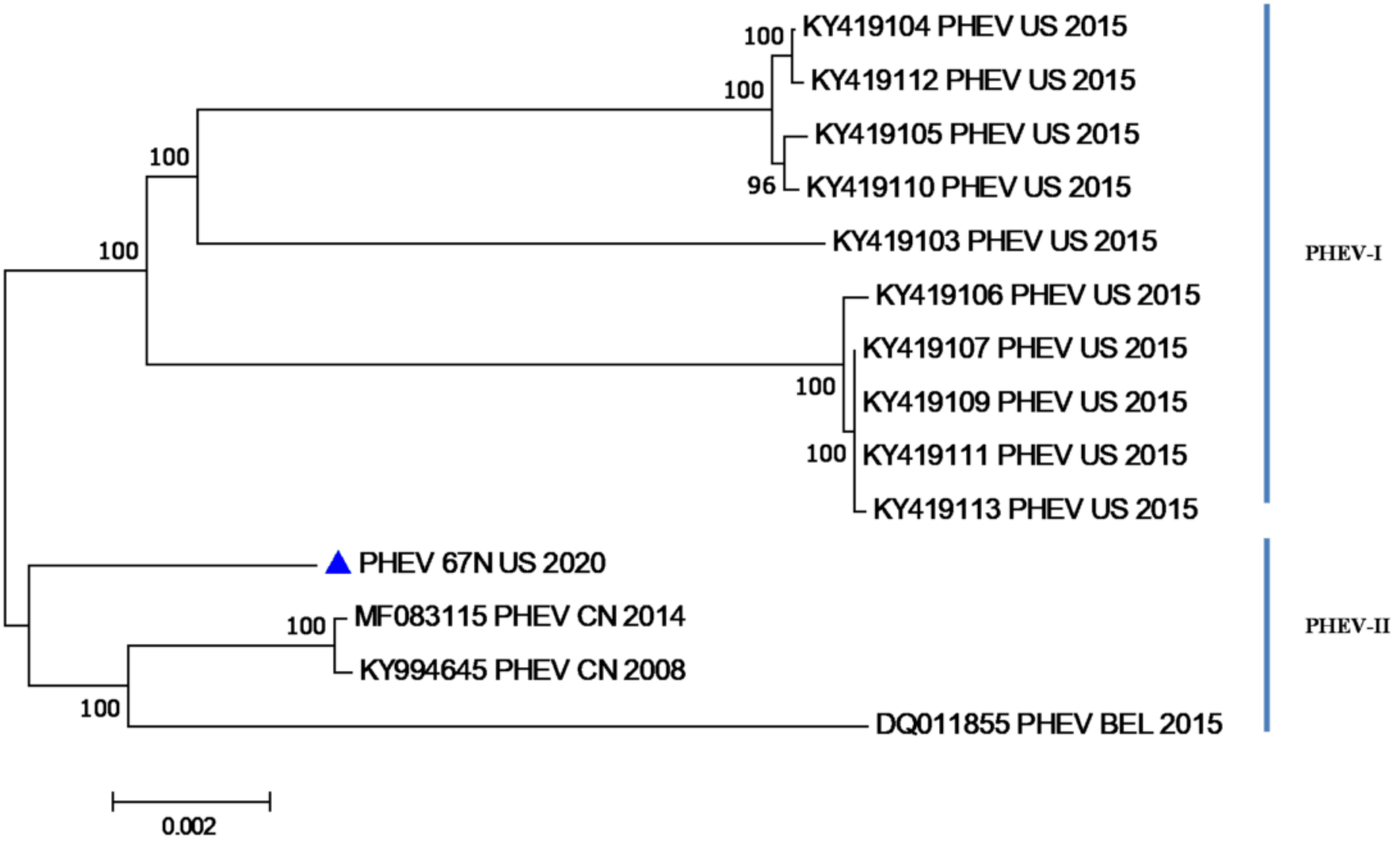


**Fig. S1** Phylogenetic analysis based on the complete genome sequences of PHEV strain 67N (PHEV/67N/US/2020) assembled in this study (blue triangle), and the 13 aditional complete sequences from different PHEV isolates available in GenBank. The phylogenetic tree was constructed by the neighbor-joining (NJ) method. Significant bootstrap values are indicated as a percentage for 1000 replicates.

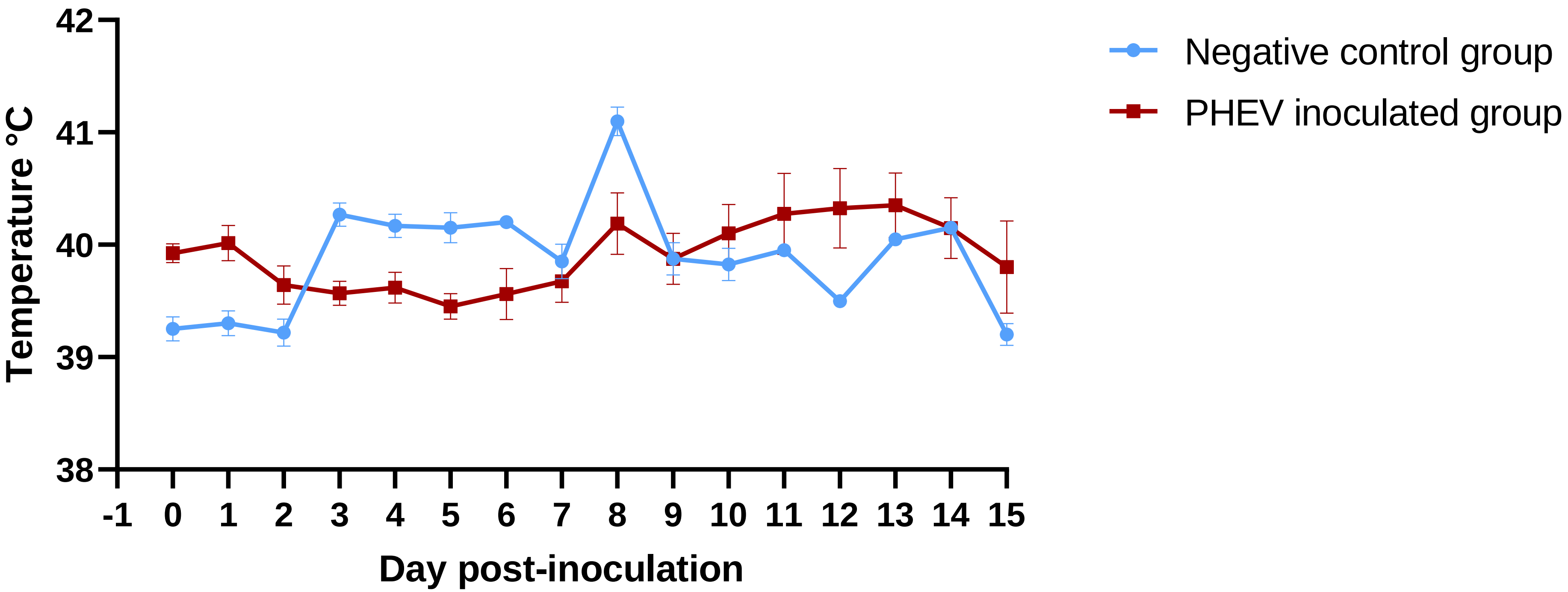
Temperature °C
42
41
40
39
38
-1
0
1
2
3
4
5
6
7
8
9
10
11
12
13
14
15
Day post-inoculation
Negative control group
PHEV inoculated group

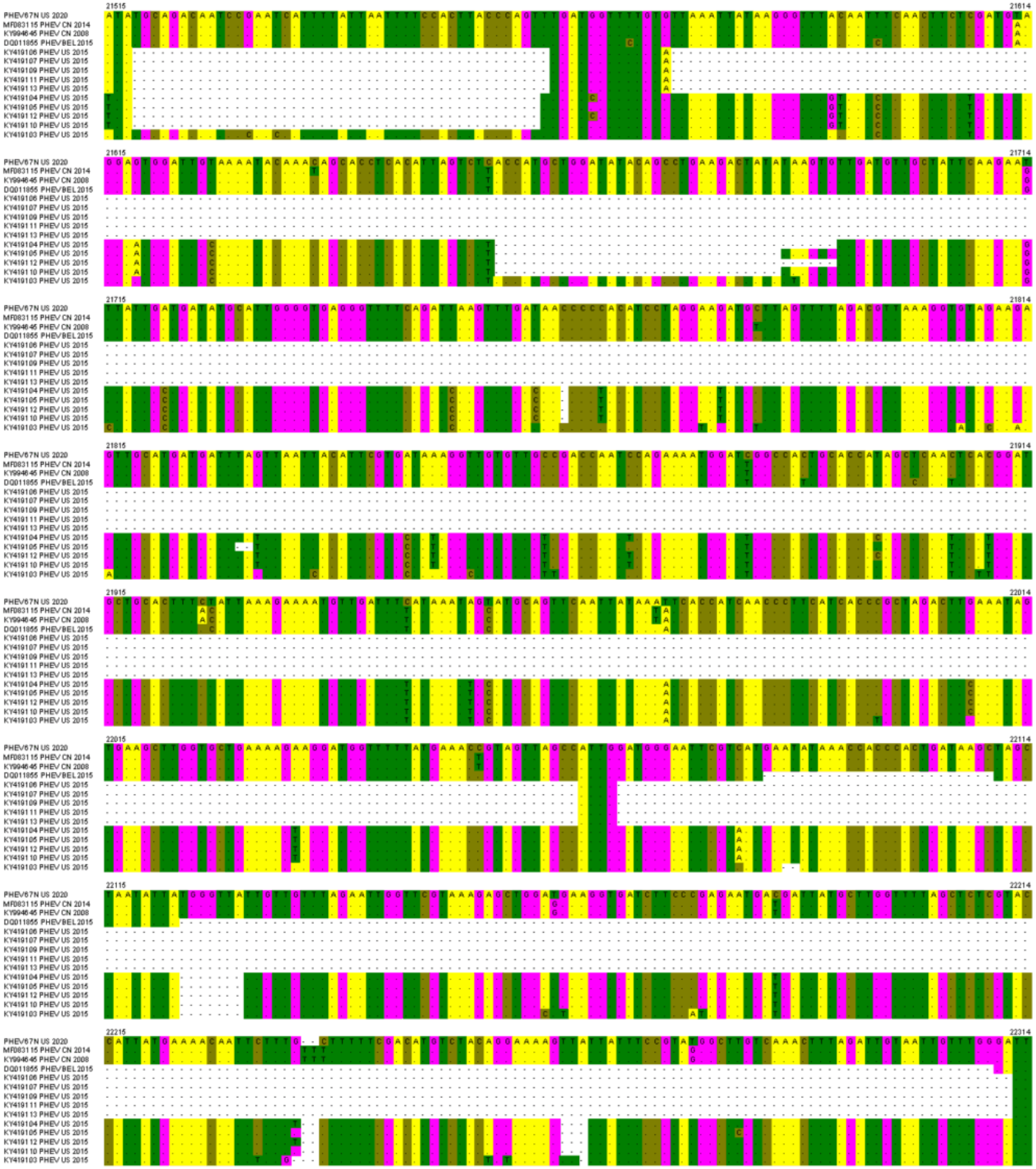
PHEV67N US 2020
MF083115 PHEV CN 2014
KY994645 PHEV CN 2008
DQ011855 PHEV BEL 2015
KY419106 PHEV US 2015
KY419107 PHEV US 2015
KY419109 PHEV US 2015
KY419111 PHEV US 2015
KY419113 PHEV US 2015
KY419104 PHEV US 2015
KY419105 PHEV US 2015
KY419112 PHEV US 2015
KY419110 PHEV US 2015
KY419103 PHEV US 2015
21515
21614
21615
21714
21715
21814
21815
21914
21915
22014
22015
22114
22115
22214
22215
22314

**Movie S1.** Cilia cell development of completely diffentiated neonatal pig-derived tracheal cells after 30 days of culture and propagation in transwells.
